# Placebo response rate in patients with chronic constipation: A protocol for systematic review

**DOI:** 10.1097/MD.0000000000019020

**Published:** 2020-02-21

**Authors:** Jie Chen, Xinghuang Liu, Tao Bai, Xiaohua Hou

**Affiliations:** Division of Gastroenterology, Union Hospital, Tongji Medical College, Huazhong University of Science and Technology, 1277 Jiefang Road, Wuhan 430022, China.

**Keywords:** chronic constipation, meta-analysis protocol, placebo response rate

## Abstract

**Objective:**

The aim of this systematic review and meta-analysis is to calculate the pooled placebo response rate in patients with chronic constipation (CC) in randomized controlled trial (RCT) and its related factors.

**Method:**

This systematic review and meta-analysis will be conducted under the guidance of Cochrane Handbook. The inclusive and exclusive criteria and search strategies for PubMed, Cochrane Library, and Embase will be introduced in this protocol. Data collection, extraction, and assessment of risk of bias will be conducted independently by 2 reviewers. The pooled placebo response rate and its 95% confidence interval (95%CI) will be calculated and the heterogeneity assessment, publication bias assessment, and subgroup analysis will be performed using R 3.6.0. This study has been registered on the PROSPERO platform (CRD42019121287).

**Result:**

The results of this systematic review and meta-analysis will be published in a peer-reviewed journal.

## 1 Introduction

Chronic constipation is a recurrent functional bowel disorder. The prevalence was estimated around 14% worldwide.^[1–5]^ Although not life threatening, it can cause the decline of patients’ quality of life and bring a heavy burden to the health service system.^[3,6,7]^ A large amount of clinical trials were conducted; however, the efficacy was still unsatisfied.^[1,3,8–10]^ Therefore, further exploring for new management remains necessary.

As it is known that most of the therapeutic randomized controlled trials (RCTs) use the placebo or sham treatment as control group, and the therapeutic effect is demonstrated through the comparison of 2 or more groups. That means placebo response rate is essential during the efficacy evaluation process for most of the therapeutic clinical trials. However, there is no article yet has reported and analysis the fact that the placebo response rate in constipated patients varies from 7% to 71%.^[4,11–14]^

Furthermore, placebo response rate is one of the necessary parameters in sample size calculation, which is of vital importance during the design of clinical trials. Taking the 2 RCT conducted by Ziegenhagen and Kruis^[15]^ and Harish et al^[16]^ for example, both of which failed to demonstrate the statistical difference between treatment group and control group. And both authors pointed out in the article that the small sample size limited the detection of an actual therapeutic effect. In fact, appropriate sample size can not only prevent the RCT studies from the low power to detect the true difference between groups, but also prevent the waste of time, money, and resources and the delay in introducing new drug.^[17,18]^

Thus, we conduct this systematic review and meta-analysis to calculate the pooled placebo response rate in patients with chronic constipation (CC) and to discuss how different characteristics in the clinical trials might affect it. This meta-analysis has been registered with ID number CRD42019121287 on the PROSPERO International Prospective Register of systematic reviews.

## 2 Methods

### 2.1 Study selection

#### 2.1.1 Inclusive criteria

a. Randomized controlled trials or crossover designed;
b. Adults (participants aged >16 years old);
c. Diagnosis of chronic constipation, functional constipation, or IBS with predominant constipation (IBS-C) based on the opinion of the clinician or specific diagnostic criteria (Rome I, II, III or IV, etc);
d. Compared pharmacological therapies (Psyllium, PEG, Chloride channel activators Lubiprostone, Guanylate cyclase C agonists Linaclotide, Prucalopride, etc) with placebo, or compared electroacupuncture and acupuncture therapies with sham stimulation;
e. The minimum treatment duration is 7 days;
f. Placebo response rate for global improvement of constipation symptoms, or improvement of frequency of bowel movements, stool consistency, etc.

#### 2.1.2 Exclusive criteria

a. Participants in the study have constipation induced by drugs, organic diseases of digestive tract, or other systemic diseases, which are confirmed by obvious and definite evidence (e.g., the result of an endoscopy, biopsy, laboratory tests, etc).
b. The study is not an original research, or is designed to be a case–control, cross-section, or cohort study.

#### 2.1.3 Outcome measurement

After a period of treatment, placebo response rates were calculated according to patient-reported information or using questionnaires based on Rome criteria and designed at the beginning of every RCT trials. The global improvement is defined as patients reporting for overall improvement or experiencing 2 or more aspects of the following symptoms:

a. increase in bowel movement;
b. reduced frequency of hard or lumpy stools;
c. reduced frequency of straining;
d. improvement of the sense of incomplete evacuation;
e. improvement of the feeling of anorectal blockage;
f. decrease of the need for digital manoeuvres to assist defecation;
g. improvement in abdominal pain (for IBS-C only).

The primary outcome is the placebo response rate for global improvement. The additional outcome is the placebo response rate for one of the above improvements.

### 2.2 Records retrieve

PubMed, Cochrane Library, Embase will be electronically searched from their inception to December 12, 2019 with no restriction of publication dates and languages. The search strategy will include both the medical subject headings (MeSH) terms and the keywords that describe the intervention (placebo, sham stimulation), characteristics of participants (chronic constipation, functional constipation, IBS-C, Fecal Impaction, Colonic Inertia), and randomized controlled trials.

#### 2.2.1 Search strategy for PubMed

#1 randomized controlled trial[Publication Type] OR controlled clinical trial[Publication Type] OR randomized[Title/Abstract] OR placebo[Title/Abstract] OR drug therapy[MeSH Terms] OR randomly[Title/Abstract] OR trial[Title/Abstract] OR trial[Title/Abstract]
#2 animals[MeSH Terms] NOT humans[MeSH Terms]
#3 #1 NOT #2
#4 chronic constipation[Title/Abstract] OR constipation[Title/Abstract] OR constipation[MeSH Terms] OR Dyschezia[Title/Abstract] OR Colonic Inertia[Title/Abstract] OR Fecal Impaction[Title/Abstract] OR impacted stool[Title/Abstract] OR lumpy stool[Title/Abstract] OR rock like stool[Title/Abstract]
#5 opioid[Title] OR cancer[Title] OR carcinoma[Title]
#6 child[Title] OR children[Title] OR childhood[Title]
#7 #3 AND #4 NOT #5 NOT #6

#### 2.2.2 Search strategy for embase (accessed via OVID)

1. randomized controlled trial.mp. or exp randomized controlled trial/
2. (random∗ or factorial∗ or crossover∗ or placebo∗).ab.
3. 1 or 2
4. chronic constipation.mp. or exp constipation/ or exp chronic constipation/
5. limit 4 to (human and(adult <18–64 years> or aged <65+ years>))
6. 3 and 5

#### 2.2.3 Search strategy for cochrane

#1 MeSH descriptor: [Randomized Controlled Trial] explode all trees
#2 randomized controlled trial
  in Trials (Word variations have been searched)
#3 #1 OR #2
#4 MeSH descriptor: [Constipation]explode all trees
#5 constipation
  in Trials (Word variations have been searched)
#6 #4 OR #5
#7 MeSH descriptor: [Placebos]explode all trees
#8 placebo
  in Trials (Word variations have been searched)
#9 #7 OR #8
#10 #3 AND #6 AND #9

### 2.3 Data collection

#### 2.3.1 Screening for eligible records

Using the search strategies as stated above, the records retrieve will be conducted independently by 2 reviewers (JC and XL) according to the Cochrane Handbook. Then 2 reviewers will independently screen both titles and abstracts for eligibility based on the inclusive and exclusive criteria described in Section 2.1. The records management is performed using EndNote X9. The detail information about this procedure will be summarized in the form of a PRISMA flow diagram.

#### 2.3.2 Data extraction and assessment of risk of bias

Full text of each eligible articles will be viewed and the related data will be extracted by 2 reviewers (JC and XL) independently according to the Cochrane Handbook. The 5-scale Jadad score (2 points for randomization, 2 points for Double blinding, and 1 point for Drop-outs or withdrawals) and the statement of allocation concealment will be used to assess the quality and the risk of bias of each studies.^[19,20]^ Any differences emerged during this procedure will be discussed by the 2 reviewers (JC and XL). If no consensus is reached, then an independent reviewer (TB) will be consulted for further solution. The data needed to be extracted includes: year, geographical location, number of centers, criteria used to define chronic constipation, active treatment, duration of therapy, dosing schedule, sample size, placebo response rate (%), etc.

#### 2.3.3 Deal with missing data

We would retrieve manuscripts from publishers, supplementary documents, corresponding records on *ClinicalTrials.gov* or contact the author for original data, if the experimental data were found to be inadequate or missing. Inadequate data would be excluded if original data cannot be retrieved.

### 2.4 Statistical analysis

#### 2.4.1 Data synthesis and heterogeneity assessment

All studies that meet the inclusion criteria and have complete data will be incorporated into the final data synthesis process. R 3.6.0 will be used to conduct all the statistical analysis, first we will calculate the pooled placebo response rate and its 95% confidence interval (95%CI) and draw the forest plot. Then the heterogeneity among all the included studies will be assessed using the *I*^2^ statistic. If it is not appropriate to conduct meta-analysis, we will then perform a systematic review only.

#### 2.4.2 Publication bias assessment

Funnel plot will be drawn to evaluate the publication bias visually, and after that the Trim and Fill of the funnel plot will be conducted if necessary. The specific test, such as Egger's test and Begg's test will also be conducted to provide more exact evidence for publication bias.^[21,22]^

#### 2.4.3 Subgroup analysis and meta-regression

If significant heterogeneity were found (*I*^2^ > 50%), the subgroup analysis and meta-regression would be performed to seek the potential reason that may cause the heterogeneity.

#### 2.4.4 Confidence in cumulative evidence

The Grading of Recommendations, Assessment, Development, and Evaluation (GRADE)^[23]^ will be used to assess the strength of the cumulative evidence independently by 2 reviewers (JC and XL). The quality of evidence (very low, low, moderate, or high) will be assessed according to the following considerations: risk of bias, consistency, directness, and publication bias.

## 3 Discussion

The placebo response rate not only acts as a standard comparative index for most of the therapeutic clinical trials, but also has significant influence on the design of RCT studies. However, the placebo response rate in constipated patients has not been fully studied based on numerous data in clinical trials. We designed the first systematic review and meta-analysis to report the pooled placebo response rate in patients with chronic constipation.

Through this study, we will obtain the size of the pooled placebo response rate and its 95%CI, as well as how it varies with different characteristics in clinical trials. Compared with the placebo response rate reported in RCT, the pooled placebo response rate obtained in this study can maintain the inadequate power in original RCTs caused by small sample size. And this may provide some references for later therapeutic clinical trials for constipation. Although the top position of systematic review and meta-analysis in the evidence pyramid has been questioned in recent years, it is no doubt that this statistical method plays a crucial role on exploring evidence based on considerable original studies.^[24,25]^ This protocol is reported under PRISMA-P.^[26]^

## Author contributions

Tao Bai and Xiaohua Hou developed the main idea of this study. Jie Chen developed the search strategy. Jie Chen and Xinghuang Liu will finish all the selection of studies, data extraction, the assessment of the risk of bias, and data synthesis. The disagreements between Jie Chen and Xinghuang Liu will be arbitrated by Tao Bai. Jie Chen drafted the original manuscript of the protocol, which was revised by Tao Bai. All authors have read and approved the final manuscript of the protocol.

Jie Chen: 0000-0002-4018-2502.

Xinghuang Liu: 0000-0001-9992-6230.

Tao Bai: 0000-0001-9697-8510.

Xiaohua Hou: 0000-0002-4777-7920.

## References

[1] Mearin F Lacy BE Chang L Bowel disorders. Gastroenterology 2016;150:1393–407. 10.1053/j.gastro.2016.02.031 27144627

[2] Camilleri M Ford AC Mawe GM Chronic constipation. Nat Rev Dis Primers 2017;3:17095. 29239347 10.1038/nrdp.2017.95

[3] Rao SS Rattanakovit K Patcharatrakul T Diagnosis and management of chronic constipation in adults. Nat Rev Gastroenterol Hepatol 2016;13:295–305. 27033126 10.1038/nrgastro.2016.53

[4] Kamm MA Muller-Lissner S Talley NJ Tegaserod for the treatment of chronic constipation: a randomized, double-blind, placebo-controlled multinational study. Am J Gastroenterol 2005;100:362–72. 15667494 10.1111/j.1572-0241.2005.40749.x

[5] Lembo AJ Kurtz CB Macdougall JE Efficacy of linaclotide for patients with chronic constipation. Gastroenterology 2010;138:886–95.e881. 20045700 10.1053/j.gastro.2009.12.050

[6] Dennison C Prasad M Lloyd A The health-related quality of life and economic burden of constipation. PharmacoEconomics 2005;23:461–76. 15896098 10.2165/00019053-200523050-00006

[7] Higgins PD Johanson JF Epidemiology of constipation in North America: a systematic review. Am J Gastroenterol 2004;99:750–9. 15089911 10.1111/j.1572-0241.2004.04114.x

[8] Liu Z Yan S Wu J Acupuncture for chronic severe functional constipation: a randomized trial. Ann Intern Med 2016;165:761–9. 27618593 10.7326/M15-3118

[9] Emmanuel AV Roy AJ Nicholls TJ Prucalopride, a systemic enterokinetic, for the treatment of constipation. Aliment Pharmacol Ther 2002;16:1347–56. 12144586 10.1046/j.1365-2036.2002.01272.x

[10] Ding C Ge X Zhang X Efficacy of synbiotics in patients with slow transit constipation: a prospective randomized trial. Nutrients 2016;8:605. 10.3390/nu8100605 PMC5083993 27690093

[11] Lacy BE Schey R Shiff SJ Linaclotide in chronic idiopathic constipation patients with moderate to severe abdominal bloating: a randomized, controlled trial. PloS One 2015;10:e0134349–1134349. 26222318 10.1371/journal.pone.0134349 PMC4519259

[12] Ibarra A Latreille-Barbier M Donazzolo Y Effects of 28-day Bifidobacterium animalis subsp. lactis HN019 supplementation on colonic transit time and gastrointestinal symptoms in adults with functional constipation: a double-blind, randomized, placebo-controlled, and dose-ranging trial. Gut Microbes 2018;9:236–51. 29227175 10.1080/19490976.2017.1412908 PMC6219592

[13] Tack J Müller-Lissner S Bytzer P A randomised controlled trial assessing the efficacy and safety of repeated tegaserod therapy in women with irritable bowel syndrome with constipation. Gut 2005;54:1707–13. 16020489 10.1136/gut.2005.070789 PMC1774790

[14] Piessevaux H Corazziari E Rey E A randomized, double-blind, placebo-controlled trial to evaluate the efficacy, safety, and tolerability of long-term treatment with prucalopride. Neurogastroenterol Motil 2015;27:805–15. 25808103 10.1111/nmo.12553

[15] Ziegenhagen DJ Kruis W Cisapride treatment of constipation-predominant irritable bowel syndrome is not superior to placebo. J Gastroenterol Hepatol 2004;19:744–9. 15209619 10.1111/j.1440-1746.2004.03384.x

[16] Harish K Hazeena K Thomas V Effect of tegaserod on colonic transit time in male patients with constipation-predominant irritable bowel syndrome. J Gastroenterol Hepatol 2007;22:1183–9. 17688659 10.1111/j.1440-1746.2006.04543.x

[17] Ebrahim Valojerdi A Tanha K Janani L Important considerations in calculating and reporting of sample size in randomized controlled trials. Med J Islamic Republic Iran 2017;31:127. 10.14196/mjiri.31.127 PMC6014761 29951427

[18] Rohrig B du Prel JB Wachtlin D Sample size calculation in clinical trials: part 13 of a series on evaluation of scientific publications. Dtsch Arztebl Int 2010;107:552–6. 20827353 10.3238/arztebl.2010.0552 PMC2933537

[19] Jadad AR Moore RA Carroll D Assessing the quality of reports of randomized clinical trials: is blinding necessary? Control Clin Trials 1996;17:1–2. 8721797 10.1016/0197-2456(95)00134-4

[20] de la Iglesia-Garcia D Huang W Szatmary P Efficacy of pancreatic enzyme replacement therapy in chronic pancreatitis: systematic review and meta-analysis. Gut 2017;66:1354–5. 10.1136/gutjnl-2016-312529 PMC5530474 27941156

[21] Sterne JA Sutton AJ Ioannidis JP Recommendations for examining and interpreting funnel plot asymmetry in meta-analyses of randomised controlled trials. BMJ 2011;343:d4002. 21784880 10.1136/bmj.d4002

[22] Peters JL Sutton Aj Fau - Jones DR Jones Dr Fau - Abrams KR Comparison of two methods to detect publication bias in meta-analysis. JAMA 2006;(1538–3598 (Electronic)). 10.1001/jama.295.6.676 16467236

[23] Guyatt GH Oxman Ad Fau - Vist GE Vist Ge Fau - Kunz R GRADE: an emerging consensus on rating quality of evidence and strength of recommendations. BMJ 20082008;336:924–6. (1756–1833 (Electronic)). 10.1136/bmj.39489.470347.AD PMC2335261 18436948

[24] Murad MH Asi N Alsawas M New evidence pyramid. Evid Based Med 2016;21:125. 27339128 10.1136/ebmed-2016-110401 PMC4975798

[25] Shaneyfelt T Pyramids are guides not rules: the evolution of the evidence pyramid. Evid Based Med 2016;21:121. 27405600 10.1136/ebmed-2016-110498

[26] Shamseer L Moher D Clarke M Preferred reporting items for systematic review and meta-analysis protocols (PRISMA-P) 2015: elaboration and explanation. BMJ 2015;350:g7647. 25555855 10.1136/bmj.g7647

